# Flexural Behavior of Natural Hybrid FRP-Strengthened RC Beams and Strain Measurements Using BOTDA

**DOI:** 10.3390/polym13203604

**Published:** 2021-10-19

**Authors:** Krisada Chaiyasarn, Nazam Ali, Phatthanayu Phuphasuwan, Nakhorn Poovarodom, Panuwat Joyklad, Hisham Mohamad, Mingliang Zhou, Qudeer Hussain

**Affiliations:** 1 Thammasat Research Unit in Infrastructure Inspection and Monitoring, Repair and Strengthening (IIMRS), Thammasat School of Engineering, Thammasat University, Rangsit, Klong Luang Pathumthani 12121, Thailand; ckrisada@engr.tu.ac.th (K.C.); phuphasuwan@gmail.com (P.P.); Poovarodom@gmail.com (N.P.); 2 Department of Civil Engineering, School of Engineering, University of Management and Technology, Lahore 54770, Pakistan; nazam.ali@umt.edu.pk; 3 Department of Civil and Environmental Engineering, Srinakharinwirot University, Nakhonnayok 26120, Thailand; 4 Civil and Environmental Engineering Department, Universiti Teknologi PETRONAS, Seri Iskandar 32610, Perak, Malaysia; hisham.mohamad@utp.edu.my; 5 Key Laboratory of Geotechnical and Underground Engineering of Ministry of Education, Department of Geotechnical Engineering, Tongji University, Siping Road 1239, Shanghai 12476, China; zhoum@tongji.edu.cn; 6 Center of Excellence in Earthquake Engineering and Vibration, Department of Civil Engineering, Chulalongkorn University, Bangkok 10330, Thailand

**Keywords:** natural hybrid FRP, BOTDA, optical fibers, natural jute FRP, basalt FRP, ATENA

## Abstract

Experimental and finite element analysis results of reinforced concrete beams under monotonic loading were presented in this study. In the experimental program, one beam was tested in an as-built condition. The other two beams were strengthened using natural hybrid FRP layers in different configurations. The natural hybrid FRP composite was developed by using natural jute FRP and basalt FRP. One of the most appealing advantages of natural fiber is its beneficial impact on the environment, which is necessary for the sustainability recognition as an alternative to synthetic FRP. The hybrid FRP was applied to the bottom concrete surface in one beam, while a U-shaped strengthening pattern was adopted for the other beam. The flexural behavior of each beam was assessed through strain measurements. Each beam was incorporated with conventional strain gages, as well as the Brillouin Optical Time Domain Analysis (BOTDA) technique. BOTDA has its exclusive advantages due to its simple system architecture, easy implementation, measurement speed, and cross-sensitivity. The experimental results revealed that the beam strengthened with the U-shaped hybrid FRP composite pattern had a better flexural response than the other counterpart beams did both in terms of peak loads and maximum bottom longitudinal steel bar strains. Beams B-01 and B-02 exhibited 20.5% and 28.4% higher energy dissipation capacities than the control beam did, respectively. The ultimate failure of the control beam was mainly due to the flexural cracks at very low loads, whereas the ultimate failure mode of FRP composite-strengthened beams was due to the rupture of the hybrid FRP composite. Further, strain measurements using BOTDA exhibited similar patterns as conventional strain gage measurements did. However, it was concluded that BOTDA measurements were substantially influenced by the bottom flexural cracks, ultimately resulting in shorter strain records than those of conventional strain gages. Nonlinear structural analysis of the beams was performed using the computer program ATENA. The analytical results for the control beam specimen showed a close match with the corresponding experimental results mainly in terms of maximum deflection. However, the analytical peak load was slightly higher than the corresponding experimental value.

## 1. Introduction

Since the start of human-made constructions, earthquakes have been causing destruction and leaving their mark. To avoid these losses, many researchers around the globe have been proposing different techniques to strengthen the resistance of constructed structures against earthquakes. There are many reasons why it is important to assess and study the performance of structures against earthquakes, as earthquakes are one of the reasons that change the social, political, and cultural fabric of society along with causing massive structural, economic, and human losses. Therefore, with the advent of different structural materials, researchers have been studying the performance of difference materials to determine their endurance against seismic catastrophes by improving the mechanical properties of structures with different strengthening techniques. Around the globe, special attention has been given to improve the performance of structures against earthquakes. Over the previous decades, many research studies have reported a significant number of modifications to the building standards to reduce the associated seismic risks. These efforts to reduce the seismic losses have become more prominent in recent years, which reflect the growing public desire to preserve the built environment against these catastrophes by using different strengthening techniques. In addition to earthquakes, there are also some other factors that lead to the degradation of concrete structures such as temperature and humidity, freeze–thaw cycle, UV irradiation, static/dynamic loading, and other coupling environments [1,2]. Therefore, it is imperative to investigate the performance of reinforced concrete beams under different configurations and using sustainable FRP composites to determine how to improve the performance of the concrete structures.

Structural strengthening and the upgradation or repairing of reinforced structures (RC) with different materials have evolved over the years and have become a complex science. This science of strengthening involves the use of different conventional cement-based materials, concrete jacketing [3,4,5], steel jacketing [6,7,8,9], as well as the use of new composite materials [10,11,12,13,14]. Regardless of the experience and experiments, the knowledge gained over the years dictates that concrete deteriorates due to natural causes and different manmade errors. However, the conventional RC and steel jacketing techniques are the cause of the significant increase in the weight of the structures and do not allow its use during strengthening, consequently causing an extra burden of increased costs and arrangements on the foundation of the structures. Further, both the steel and concrete jacketing alter the stiffness of the member with steel jackets being further prone to corrosion [15,16]. Therefore, in recent years, the use of Fiber-Reinforced Polymer (FRP) composites has gained much popularity because they do not significantly increase the weight of the structures and are easy to apply, which greatly improve the bearing capacities of the component members and enable the use of structures during strengthening [17,18,19,20].

Due to the convenience of application and short time required for the application of FRP composites, they are becoming an effective strengthening substitute. The reinforcement of structural members with the help of different FRP composites using different configurations and types has been studied by many researchers. The use of glass and basalt fiber for the strengthening of concrete structures at different temperatures was investigated, and it was found that basalt fiber performed better than glass fiber. Basalt fiber outperformed in flexural strength testing; both the yielding and ultimate strength of the specimen improved up to 27% depending upon the application of the number of layers [21]. The performance of natural hemp fiber was tested to determine the flexural capacity of the unreinforced masonry walls, and it was inferred from a sensitivity analysis that the flexural capacity and ductility of the masonry structures increased with the reinforced ratio [22]. Sisal fibers were used for the reinforced cementitious strengthening of masonry structures, and it was reported that with the application of loading, the stiffness effect of mortar between cracks progressively reduced compared with reference masonry structures [23,24,25]. Based on the performance and properties of these different types of fibers, different researchers have studied their impacts and reported their findings. For example, it has been reported that the basalt fibers have better tensile strength as compared with the glass fibers, greater failure strain than the carbon fibers, and good resistance to chemical attack [26]. Due to these advantages, the use of basalt fibers for the applicability of structural strengthening is more and highly expected. The behavior of hemp and jute fiber is more brittle as compared with basalt fiber, while the basalt fiber has a higher strain failure than jute fiber [14].

It has been observed that synthetic FRP composites have high strength and low weight and are widely used in building construction. Some of the synthetic FRP composites include carbon, glass, aramid, or basalt fibers, which are externally bonded to increase the stiffness, load carrying, and resistance to environmental corrosion. However, the manufacturing process of these synthetic FRP composites consumes a lot of energy, which poses environmental threats to the eco-system after they are wasted. Therefore, due to the increased recognition of climate change, natural fibers have become an alternative and attractive element of strengthening as compared with the synthetic FRP composite. One of the most compelling benefits of using natural fibers is their sustainability to the environment. Another benefit of the natural fibers is the low cost incurred for their manufacturing process [27,28]. The cost-efficiency of the natural fibers has been extensively studied by many researchers. For example, it was stated that the cost efficiency of jute fiber is 20–50% as compared to the glass fiber, which is capable of resisting the tensile load of 100 kN [29]. Though synthetic FRP composites have the advantage of high tensile strength, they are not environmentally sustainable, which makes them less acceptable as a strengthening element in building construction. On the contrary, the disadvantages of natural fibers include the lower tensile strength, poor durability once exposed to moisture, and large scatter in the material properties. Additionally, there is some uncertainty in the literature regarding the cost-efficiency of the natural fibers, which mostly comprise the cost of the raw material and does not include the manufacturing cost. Additionally, the size effect is neglected because when natural fibers are used in larger quantities for flexural strengthening, they underperform as compared with the synthetic FRP composite. Therefore, it is necessary and imperative to investigate and evaluate the combined performance of synthetic and natural fibers to comprehend their performance in building construction. Different researchers have used different combinations of the hybrid FRP composite such as palm/kenaf, basalt/biocarbon, banana/glass, and wood/glass [14,17]. These different combinations help in changing the properties of different composite applications [30]. Some of the common benefits of using these hybrid FRP composites can be attributed to their reduced costs in production, better chemical resistance, improved mechanical properties, and high thermal stability. However, it is very important to find a balance in the properties of the composite materials in order to attain the required properties of the materials.

In this research study, combined/hybrid FRP composites were used to investigate the performance of beams, as it is evident from the literature that natural fibers are more sustainable but exhibit lower performance in flexural strengthening as compared with synthetic fibers, which are strong in flexural behavior but exhibit brittle failure. Therefore, in this study, the combined behavior of natural fiber (jute) and synthetic fiber (basalt) was studied on beams. Additionally, the performance of the failure behavior of these beams was monitored using two techniques, including conventional strain gauges and Brillouin Optical Time Domain Analysis (BOTDA). Different research studies have revealed that the sensitivity of strain gauges is higher as compared with BODTA, but they are costly and difficult to monitor because of the complexity of data logging. In addition, strain gauges monitor the local failure behavior, while BODTA is used for monitoring global failure behavior. The costs incurred in the BODTA technique are lower than those of the strain gauges.

This study investigated the effectiveness of composite natural jute and basalt fibers in the flexural strengthening of RC beams. This hybrid composite scheme employed the strengths of each fiber to overcome the weaknesses of the other fiber. To the authors’ knowledge, this hybrid scheme has not been employed in the past. Further, the efficiency of optical fiber strain sensing (BOTDA) was assessed with conventional strain gage readings. This paper is organized as follows: Section 2 describes the experimental program, materials, and methods used in this study. Section 3 discusses the results and main findings of this research study. Detailed discussion on the results and some of the findings are described in Section 4. Finally, Section 4 summarizes the conclusions, and future research directions are also proposed.

## 2. Materials and Methods

**Figure 1:**
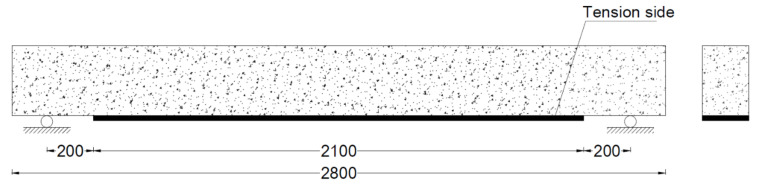
Strengthening detail of beam B-01 (units: mm).

**Figure 2:**
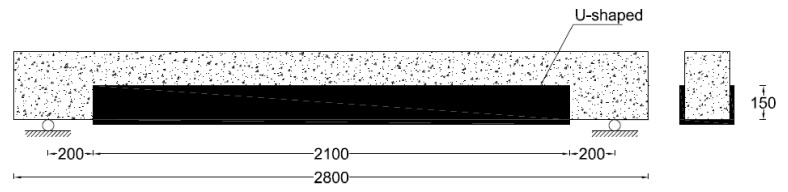
Strengthening detail of beam B-02 (units: mm).

A total of 3 beams were tested in this study. One beam was tested without strengthening and was referred to as the control beam. The other 2 beams were strengthened using hybrid natural jute and basalt FRPs. Two layers each of natural jute and basalt fiber were applied to each of the strengthened beams. The natural jute FRP has a lower fracture strain as compared to the basalt FRP composite. Therefore, the first two layers of the basalt FRP for strengthening was chosen. Natural jute was applied as the second two layers. The strengthening pattern of the two beams was different. On one beam, FRP layers were applied to the bottom side only, as shown in Figure 1. In previous studies, it was found that the use of FRP in the form of a u-shape i.e., at the bottom and sides (below the neutral axis), is very effective to further enhance the load carrying capacity of the RC beams as compared to the bottom side only. She et al. reported that the use of a U-shaped FRP is also very helpful to avoid the de-bonding of the FRP from the tensions side of RC beams. Therefore, in this study, the RC beam (B-02) was strengthened with a u-shaped pattern on the surface below its neutral axis, as shown in Figure 2. In the u-shaped pattern, the hybrid FRP composite was applied at the sides and bottom. Table 1 summarizes the strengthening scheme adopted in this study.

### 2.1. Specimen Details

**Figure 3:**
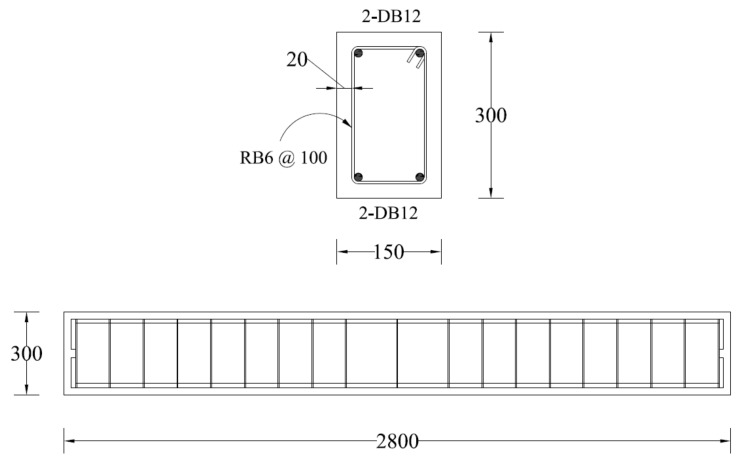
Specimen details (units: mm).

RC beams had a cross-section of 150 mm × 300 mm with a support-to-support length of 2500 mm. The total length of each beam was 2800 mm. The top and bottom longitudinal bars consisted of two 12 mm-diameter deformed bars. Shear reinforcement consisted of 6 mm diameter round bars. Within the shear span, the spacing of stirrups was 100 mm, which was doubled just outside the shear spans. A concrete cover of 20 mm was provided on all sides. Details of the RC beams are shown in Figure 3.

### 2.2. Material Properties

Deformed and plain steel bars were used for longitudinal and transverse reinforcement, respectively. Their mechanical properties were found following the protocols of ASTM A615/A615M - 20 [31]. A total number of five steel bars were tested for each type of steel bar. Table 2 presents the “average mechanical properties of steel bars” in terms of diameter, elastic modulus, yield stress, yield strain, fracture stress, and strain. All beams were constructed using a single batch of concrete. Standard cylinders were cast as per the recommendations of ASTM C39/C39M - 21 [32]. For this purpose, three cylinders of standard size, i.e., 150 mm × 300 mm (diameter × height), were cast and tested under axial compression. Table 3 shows the “average concrete characteristics.” In this study, woven basalt fabric was provided by Kamenny Vek, Russia, and locally available woven jute fabric was used. The epoxy resin was obtained from Smart and Bright Co., Ltd., Thailand. The epoxy resin was made of two parts, i.e., resin and hardener. The mixing ratio of resin was considered as 1:2 (hardener:resin). Further, the properties of FRP composites were determined following the procedures of ASTM D7565/D7565M - 10(2017) [33]. A total number of 10 tensile strips were tested to obtain the average mechanical properties of basalt and jute FRP composites. The properties of FRP composites are given in Table 4.

### 2.3. Instrumentation and Load Setup

**Figure 4:**
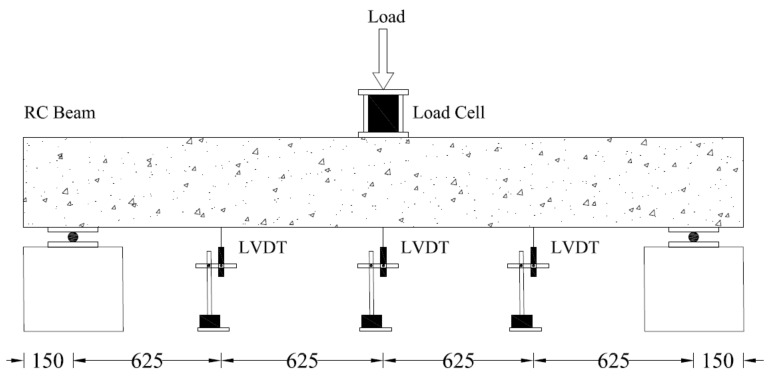
Test setup and LVDT placement (units: mm).

**Figure 5:**
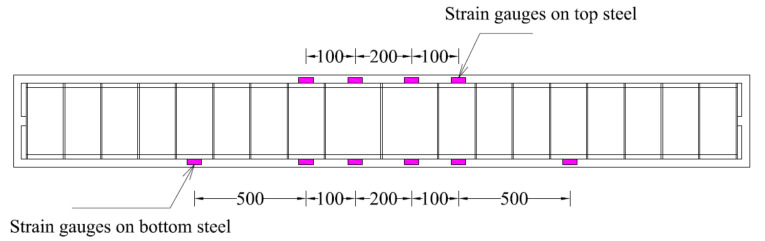
Position of strain gages on longitudinal reinforcement (units: mm).

Each beam was subjected to the four-point bending test with a load increment of 5 kN until failure. Points of load were 250 mm on each side of the centerline of the beam, as shown in Figure 4. Strain gages were installed on the bottom longitudinal bars at three different locations, as shown in Figure 5.

Four 5 mm-strain gages were mounted on the top longitudinal bars, while 6 5 mm-strain gages monitored the strains of the bottom longitudinal bars. The vertical deflection of the beams was monitored using Linear Variable Displacement Transducers (LVDTs). Four LVDTs were mounted on each beam. Two LVDTs were mounted at the beam midspan. One LVDT each was mounted at 700 mm on either side of the beam midspan.

**Figure 6:**
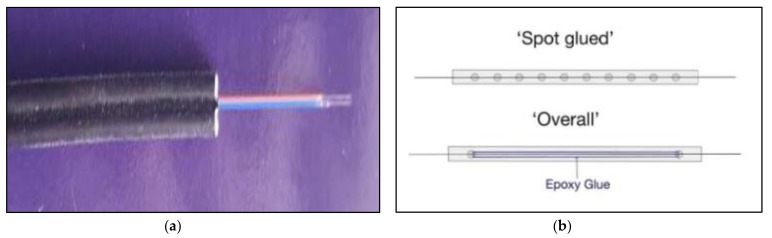
(**a**) Optical fiber used; (**b**) bonding methods.

**Figure 7:**
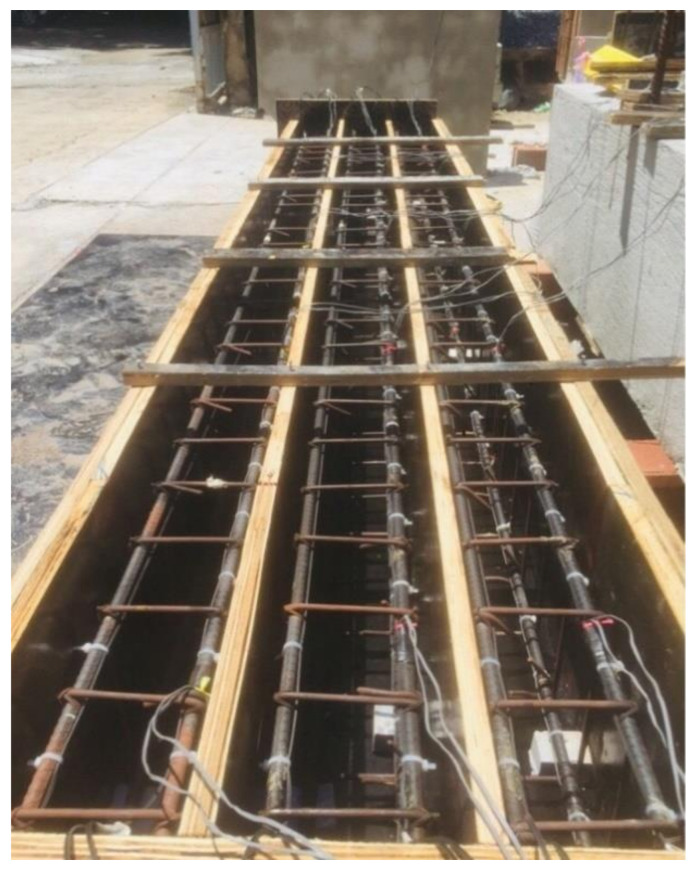
Application of spot glue for optical fiber bonding.

Multi-mode optical fibers were used as an alternative strain measuring instrument. The optical fiber was a product of Shenzhen Owire Communication Technology CO., LTD, Zhangbei Industrial Park, Longgang, Shenzhen, China. The core of the optical fiber was 9 microns, which was embedded in a glass cladding with a diameter of 125 microns, as shown in Figure 6a. There are two methods to attach the optical fiber to the system. The first is to use epoxy all along its length, as shown in Figure 6b. This method is time-consuming and takes a lot of epoxies. The second method involves the application of spot clamps at discrete points along the length of the optical fiber. The latter method was adopted in this study. Further, two configurations of spot clamps were implemented. For the first configuration, the optical fiber was spot-clamped at each interval of the stirrups, as depicted in Figure 7, and hereby referred to as spot-clamping. The second configuration involved the application of spot clamps only at the ends of the longitudinal bars (see Figure 7) and are referred to as end-clamping. Before the application of optical fibers, a tensile strain of 1000–1500 microns was applied. This helped to facilitate the reading of the data on the monitor [34]. However, the optical fiber was fully attached to the concrete surface by using the first method, which includes the application of epoxy resin all along the length of the specimen because clamps cannot be attached on the concrete surface.

**Figure 8:**
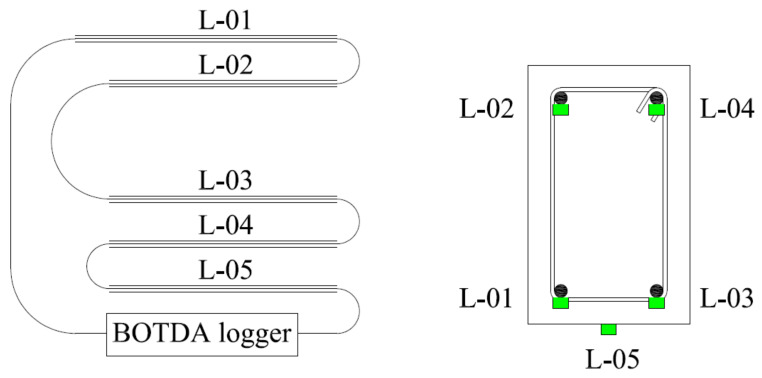
Schematic of optical fiber mounting.

**Figure 9:**
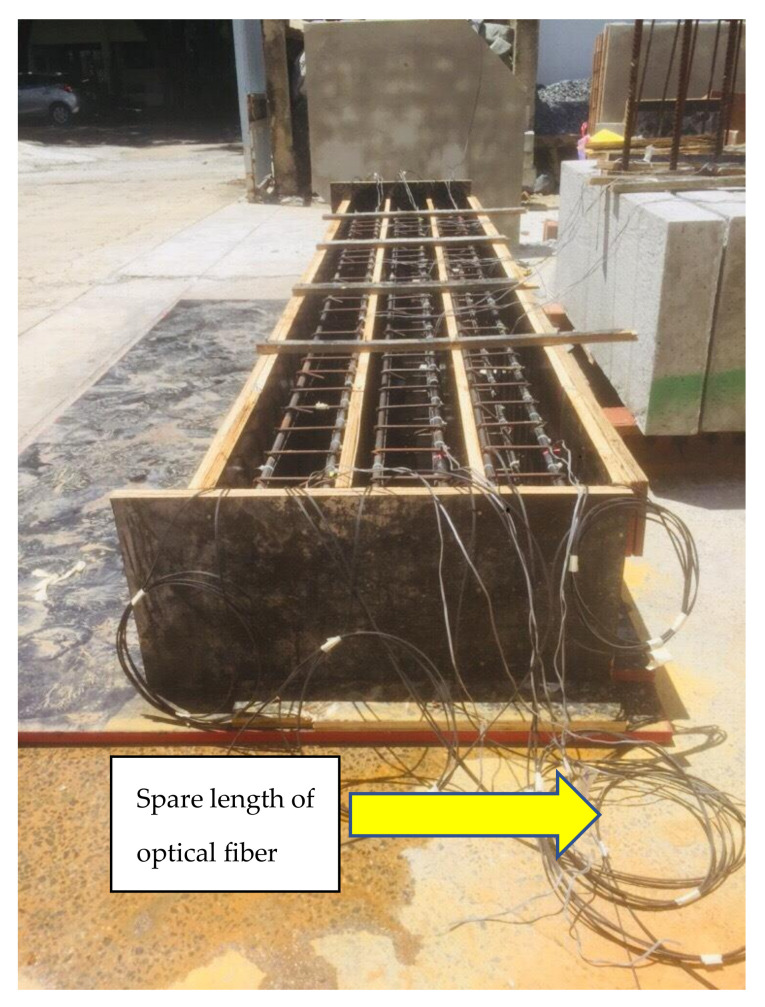
Spare length provided in optical fiber after each attachment to steel bar.

It should be mentioned that a single continuous optical fiber was used for the top and bottom reinforcement and beam bottom surface in continuation (see Figure 8). Optical fiber was mounted to the concrete surface in the case of the control beam, while it monitored strains of the FRPs in the case of strengthened specimens. The mounting sequence of optical fibers was as follows: starting from the BOTDA logger to the bottom left steel bar (L-01) to the top left steel bar (L-02) to the bottom right steel bar (L-03) to the top right steel bar (L-04) to the bottom concrete surface (L-05) before finally reaching the BOTDA logger. This sequence of optical fiber instrumentation was chosen to facilitate the application of both clamping configurations in each beam. For instance, the left bottom and top longitudinal bars were instrumented with optical fibers using the 1st configuration, while the 2nd configuration was adopted for the right bottom and top longitudinal bars. It is worth mentioning that the strain data obtained from the optical fibers were in spatial coordinates of the optical fiber. Therefore, the records of each bar were differentiated from the other two consecutive (previous and next) bars by providing a spare length of optical fiber for approximately 3 m before attaching to the next steel bar, as shown in Figure 9.

## 3. Results and Discussions

### 3.1. Failure Modes

#### 3.1.1. Beam B-Con

**Figure 10:**
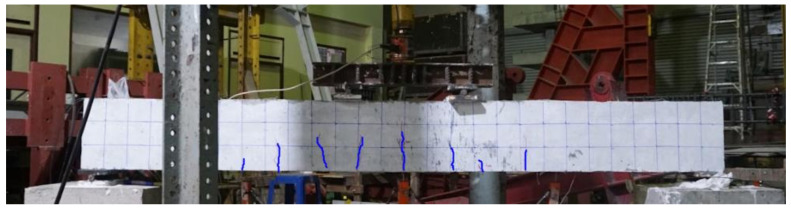
Onset of flexure cracks at early load stage.

**Figure 11:**
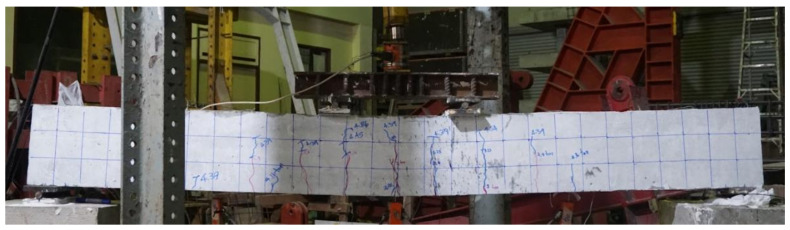
Final failure of control beam.

**Figure 12:**
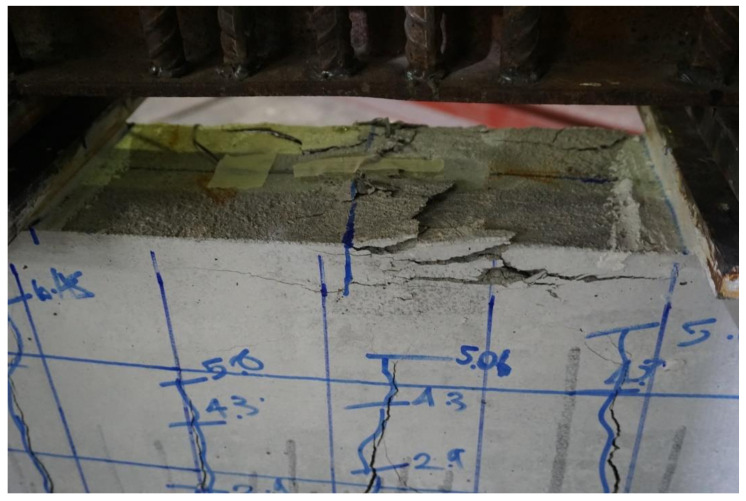
Typical crushing of concrete in all specimens.

Due to sufficient shear spans, the behavior of the control beam was controlled by flexure. Flexural cracks were observed at very low loads, as shown in Figure 10. However, this was merely a transition from the uncracked to cracked concrete stage with no drop in strength. A further increase in load accompanied the spread and generation of new flexural cracks. Failure of the control beam was observed at a 53 kN load, exhibiting large flexural cracks (see Figure 11), as well as yielding of the bottom longitudinal steel bars and crushing of the concrete at extreme compression (see Figure 12). Overall, the failure mode of beam B-Con was controlled by the tensile behavior of the longitudinal reinforcement at the tension face after the appearance of the first crack. Similar failure modes have been reported in previous studies [35,36].

#### 3.1.2. Beam B-01

**Figure 13:**
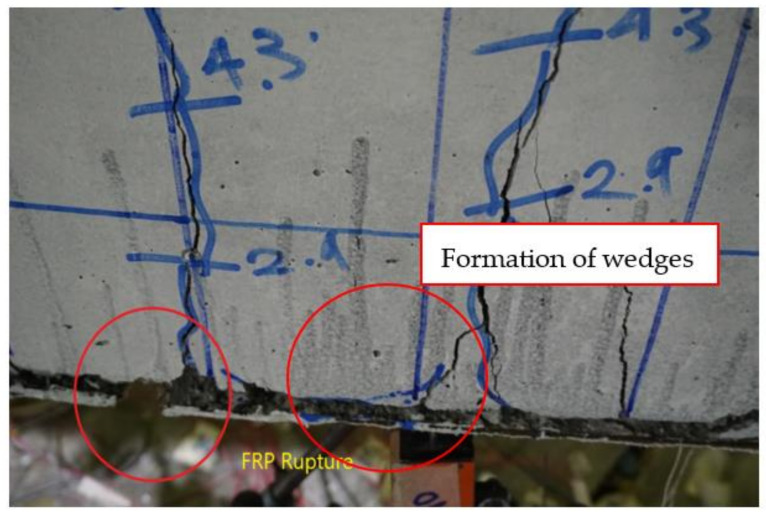
FRP rupture and wedge formation at final failure of beam B-01.

Beam B-01 also exhibited hairline flexural cracks at the early load stage. This beam failed at a 66 kN load, exhibiting large flexural cracks and yielding of longitudinal reinforcement. Unlike the control specimen, B-01 exhibited concrete compression. At failure load, rupture of the FRP was observed, reflecting that the capacity of the FRP composite was exhausted. Flexural cracks formed a wedge-shaped pattern within the vicinity of the FRP rupture, as shown in Figure 13. The formation of a wedge-shaped pattern was mainly due to the presence of the FRP composite as the tension side. Due to the FRP composite, the crack width of the flexural cracks was small and there were few cracks with a large crack width at the location of the FRP rupture. Further, FRP de-bonding was observed slightly prior to its rupture.

#### 3.1.3. Beam B-02

**Figure 14:**
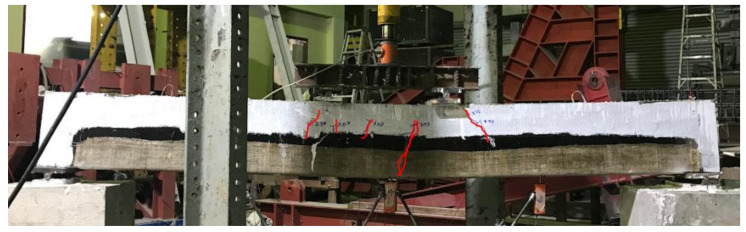
Final failure of specimen B-02.

**Figure 15:**
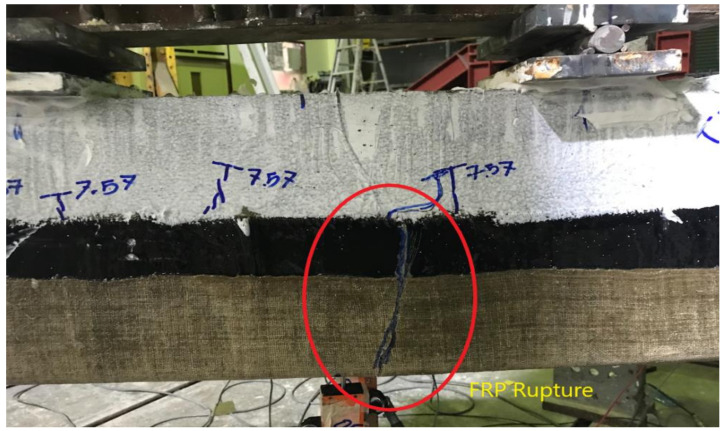
FRP rupture at failure of beam B-02.

The formation of flexural cracks at the early load stage could not be observed, due to the application of the U-shaped FRP composite layers. However, flexural cracks penetrated through the top edges of the U-shaped FRP at a failure load of 74 kN, as shown in Figure 14. No debonding of FRP was observed in contrast to the specimen B-01. However, final failure was still accompanied by FRP rupture, as shown in Figure 15.

Strain measurements revealed that strains of the bottom longitudinal bars were sufficiently exceeded beyond their yield limits. Similar to other specimens, concrete crushing was also observed at the top surface.

### 3.2. Load–Deflection Curves

**Figure 16:**
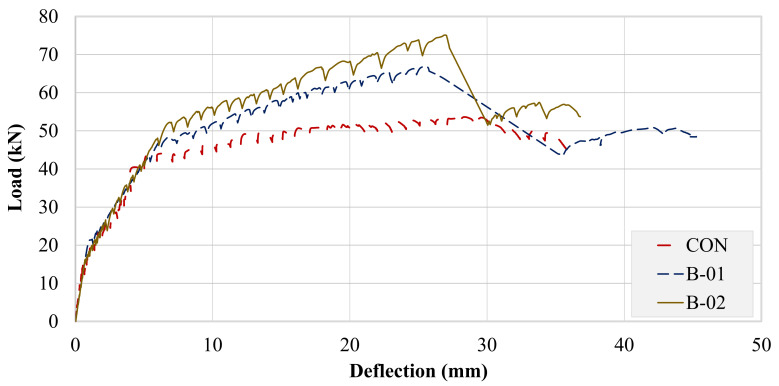
Experimental load–deflection response.

A comparison of the load–deflection curve was necessary to reveal the beneficial impact of the strengthening schemes. LVDTs were mounted at the midspan for this purpose. Figure 16 shows the measured load–deflection response of all beams. The load versus deflection response of the control beams was observed to be tri-linear. The first part represented a linear increase in the load until the first tension crack. The second part was also linear until the yielding of the steel bars. However, the stiffness of the second part was lower than that of the first part. In the third part, the load versus deflection curve was almost a straight line with a small increase in the load. In the FRP-strengthened beams, the first and second parts of the load versus deflection curves were similar to the control beam; however, in the third part, the increase in load was high as compared to the control beam due to the presence of the FRP composite at the tension side. Once the beams B-01 and B-02 reached their ultimate load, FRP rupture occurred and a sudden drop in the load versus deflection curves was observed. The sudden drop was further stabilized due to the presence of the steel bars in the tension zone of beams B-01 and B-02. It is evident that the control specimen made the lowest bound in terms of flexural strength followed by beams B-01 and B-02. Beyond the yielding point, B-Con exhibited negligible stiffness. With the concrete below the neutral axis cracked, all of the load was carried by the bottom longitudinal bars. On the contrary, both the strengthened specimens exhibited a higher post-yield stiffness than did the control specimen attributed to the support imparted by FRP layers. It can be observed that the U-shaped pattern performed better both in terms of post-yield stiffness and peak load than B-01 did. The maximum load sustained by the control specimen was 53 kN, which increased to 24.5 and 39.6% for beams B-01 and B-02, respectively.

### 3.3. Energy Dissipation Capacities (EDC)

High-energy dissipation is an indicator of the ductile mode of failure. EDC was calculated for each beam by the summation of areas under their respective load–deflection curves up to the softening point. Corresponding EDC values are tabulated in Table 5. The lowest bound of EDC was created by the control beam. On the contrary, beams B-01 and B-02 exhibited 20.5% and 28.4% higher energy dissipation capacities than the control beam did, respectively.

### 3.4. Strain Measurements of Steel Bars

#### 3.4.1. Strain Gauge Data

**Figure 17:**
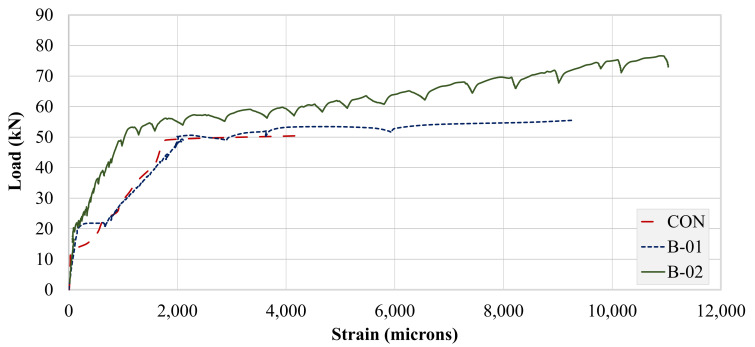
Strain gage data for bottom longitudinal bars.

Strains of the longitudinal top and bottom steel were captured using 5 mm-gage strain gages. Strain data for the bottom longitudinal bars of all beams are presented in Figure 17. Strains corresponding to yield and maximum values are tabulated in Table 6. A clear improvement in strain behavior can be observed in both the strengthened beams. The control beam failed at a very low maximum strain in comparison to the strengthened beams. The behaviors of beams B-CON and B-01 were similar up to the maximum strain recorded for beam B-CON. However, beam B-01 registered a significantly higher maximum longitudinal bar strain than that of beam B-CON. It should be mentioned that the bottom longitudinal bars of both B-CON and B-01 yielded at close strain values with a slightly lower value for beam B-01. The application of FRP layers in the U-shape resulted in a much-improved strain response than that for the FRP attached to the bottom side only. This improvement agreed with the load–deflection curves presented in earlier sections. Strengthening with FRP layers substantially reduced yield strains of the bottom longitudinal bars. This may be attributed to the inherent elastic behavior of FRP layers with additional stiffness imparted to the beams. The observed trends are in parallel with existing studies [37,38].

**Figure 18:**
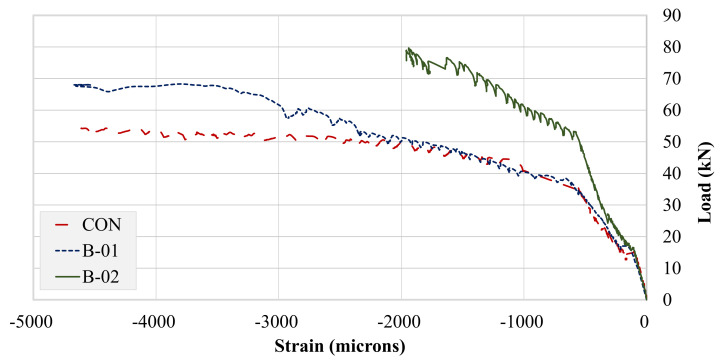
Strain gage data for top longitudinal bars.

Strain gage compression steel recordings are presented in Figure 18. A reverse trend was observed from tensile steel strain recordings. Both B-CON and B-01 recorded very high compression strain values. However, beam B-01 compression strains were limited in contrast to its tensile longitudinal strains. This is an indication of a much-improved tensile steel performance in specimen B-02. The application of U-shaped FRP layers significantly reduced the strain demand on compression steel. Further, the maximum strain obtained in beam B-02 occurred at a much higher load than that of beams B-C0N and B-01 did (see Table 6).

#### 3.4.2. BOTDA Data

**Figure 19:**
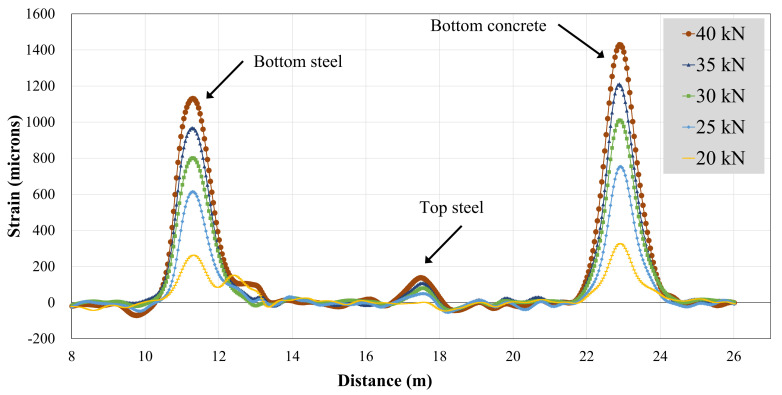
BOTDA records for beam B-CON.

**Figure 20:**
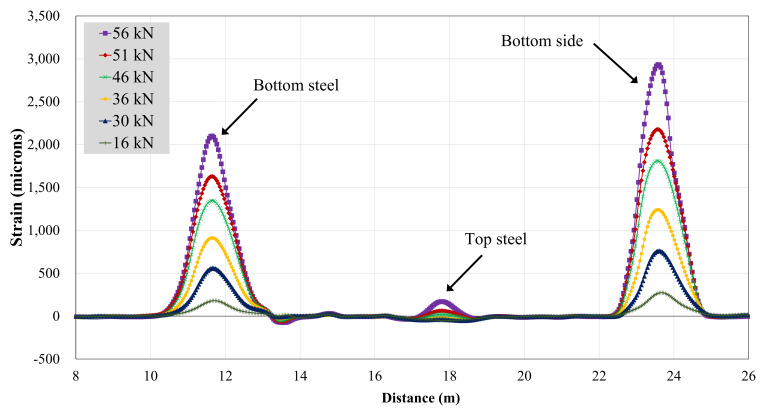
BOTDA records for beam B-02.

A single optical fiber was run along the top and bottom steel bars, as well as the concrete bottom surface. Therefore, the strain data obtained were in spatial coordinates of the optical fiber. Figure 19 and Figure 20 show strain records obtained for beams B-CON and B-02. As described in earlier sections, optical fibers were mounted to steel bars using spot and end clamping. However, the end clamping failed earlier in all three specimens, resulting in no record. Therefore, the data presented hereby only describe spot clamping records. Further, strain data could not be recorded for beam B-01, due to the malfunction of the optical fiber. Table 7 specifies maximum BOTDA strains for B-CON and B-02. Similar to strain gage recordings, the maximum strain of the control beam occurred at a lower peak load than that of beam B-02. The maximum strain of the bottom longitudinal bar was also higher in beam B-02 than B-CON. This agrees well with strain gage records of the respective beams.

#### 3.4.3. Comparison of Strain Records

**Figure 21:**
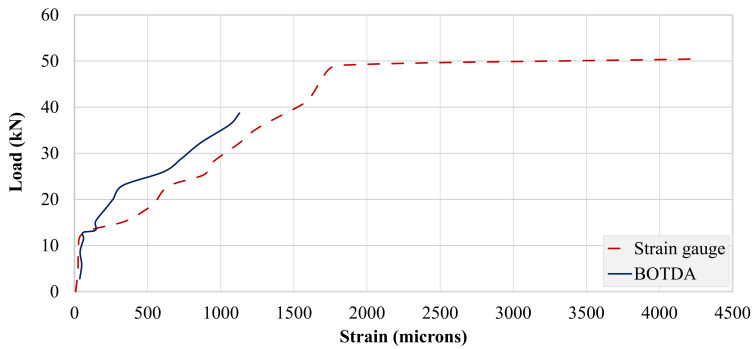
Comparison of strain records from strain gages and BOTDA for beam B-CON.

A comparison of strains obtained from strain gages and BOTDA is presented in this section. Figure 21 presents this comparison for the control beam. Up to the cracking load, both curves exhibited similar patterns. Beyond the cracking load, BOTDA records deviated from strain gage records. A further increase in load resulted in the propagation of bottom flexural cracks toward the top. The BOTDA records obtained were much shorter than the strain gage records, as optical fibers failed before the yielding of steel bars. The strain gauges usually comprised small gauge lengths, and strain measurements were local for a small part of the steel bar and/or concrete surface, whereas the BOTDA wire was continuous and installed along the full length of the steel bars. As a result, the strain monitoring through strain gauges was usually higher than that of the BOTDA. It is also believed that the upward propagation of cracks beyond the position of optical fibers might have broken them.

**Figure 22:**
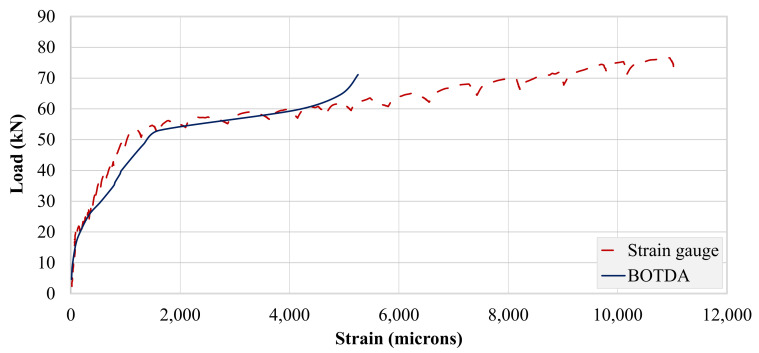
Comparison of strain records from strain gages and BOTDA for beam B-02.

This comparison for beam B-02 is presented in Figure 22. Interestingly, both curves exhibited a similar response up to the failure of optical fibers. This may be attributed to the delayed propagation and lower number of flexural cracks in beam B-02 due to the U-shaped FRP layers. This agrees with the failure mode of beam B-02 presented earlier. This delayed propagation allowed the steel bars to yield before significant cracks damaged optical fibers. Similar findings have also been found in previous studies [39,40,41].

### 3.5. Finite Element Model

**Figure 23:**
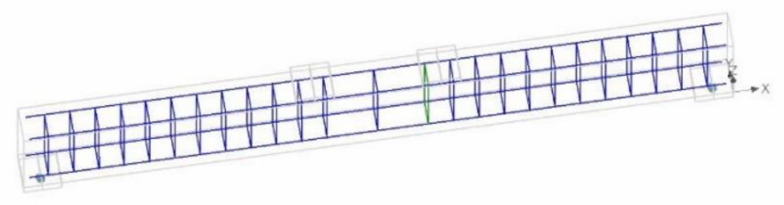
RC beam model in ATENA.

**Figure 24:**
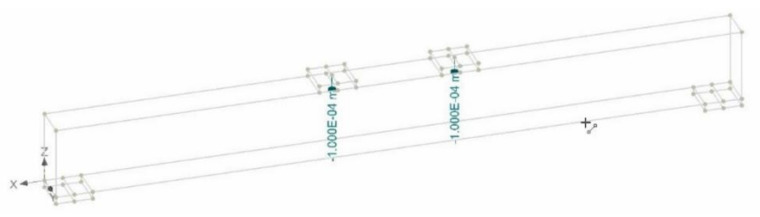
Point of application of prescribed displacement-controlled loading.

Further investigations into the structural behavior were carried out using nonlinear finite element analysis using computer program ATENA. ATENA is a tool for nonlinear structural analysis [42,43,44]. Some researchers have also used the techniques of Steel-Reinforced Grout (SRG) for the experimental investigation and modeling of reinforced concretes using different continuous and discontinuous U-shaped SRG strips. Their analytical FEM modeling predictions of the shear capacity of SRG beams are in agreement with the experimental results with an accurate average value of 0.98 of predicted/experimental ratio [45,46]. On the similar patterns, in this research study, a nonlinear FEM model was created for all three beams using the built-in material models of ATENA. Concrete was modeled using the fracture-plastic constitutive material model that is available in ATENA’s library as CC3DNonLinCementitiois2. This model accounts for the nonlinearity in both tension and compression as per the recommendations of MC10. CEB-FIP Model Code 2010 [47]. Post-cracking tensile behavior was simulated using a fictitious crack model based upon crack-opening law and fracture energy. The function of crack opening based on the exponential function experimentally derived by Hordijk 1991 [48] was utilized. Steel reinforcement was simulated using 1D truss element CC Reinforcement, which is also available in ATENA’s built-in library. As the reinforcing bars were continuous with sufficient anchorage capacities, no slip between reinforcing bars and concrete was expected. Therefore, a perfect bond between steel bars and surrounding concrete was adopted in this study. Further, the buckling of steel bars was also not considered and the same material model was used for tension and compression steel. The effect of external FRP composite sheets was considered by an elastic steel plate attached to the beam at the position of the FRP composite sheets. Figure 23 is a typical model of beams in this study, while Figure 24 shows the application of the prescribed displacement control loading.

**Figure 25:**
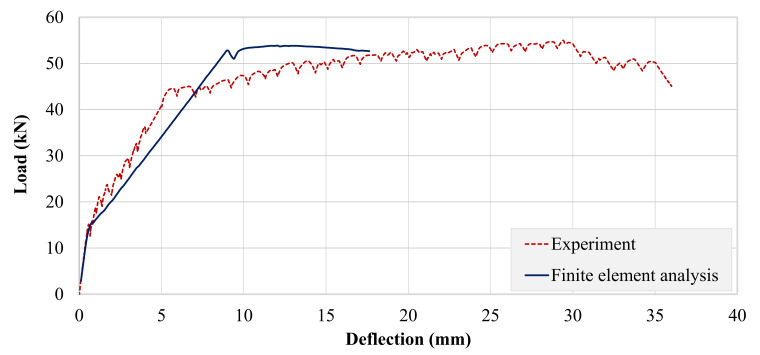
Comparison of experimental and analytical load–deflection response of control beam.

Figure 25 compares the experimental and analytical load–deflection curves of the control beam. Both curves showed similar pre-cracking stiffness and cracking loads. The experimental post-cracking stiffness was found to be higher than that obtained from ATENA. The ultimate deflection of both curves was comparable. However, the peak load obtained from analysis was higher than the experimental one.

**Figure 26:**
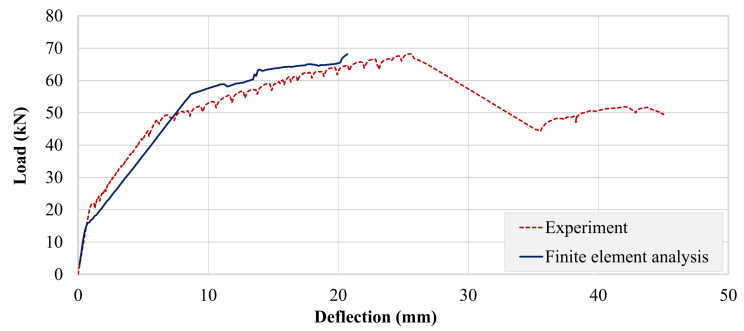
Comparison of experimental and analytical load–deflection response of beam B-01.

**Figure 27:**
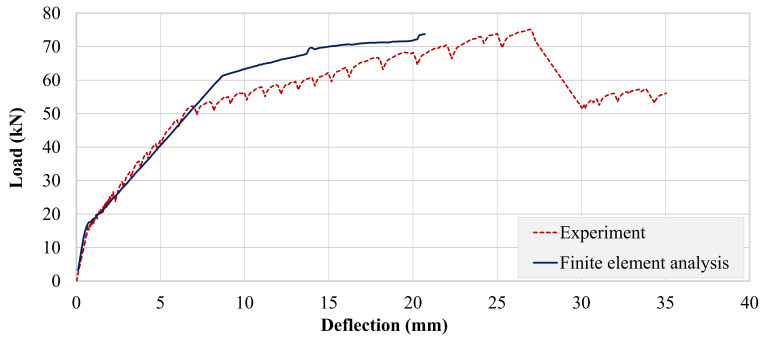
Comparison of experimental and analytical load–deflection response of beam B-02.

Experimental and analytical load–deflection curves of beam B-01 are presented in Figure 26. Here, a similar trend as that of the control beam was observed. However, analytical failure of B-01 occurred much earlier than that of the control beam. The analytical response of beam B-02 was comparable with the experimental response (see Figure 27). Post-cracking stiffness of the experimental and analytical curves was comparable, contrary to beams B-CON and B-01. However, its analytical failure occurred at a lower deflection, similar to beam B-01. In terms of peak loads, a close match between experimental and analytical values was observed for both the strengthened beams, as can be seen in Table 8. Similar trends have been also reported by Olteanu et al. 2011 [49] and Chaimahawan et al. 2021 [50].

## 4. Conclusions

This paper presented an experimental study on the flexural strengthening of RC beams using hybrid FRP composite sheets. Three beams were tested: one in an as-built condition (B-CON), one strengthened with the hybrid FRP composite on its bottom surface only, and one with the hybrid FRP composite in a U-shaped pattern applied to its tension zone. Further, the flexural response of each beam was assessed through strain measurements. The strains of the top and bottom longitudinal steel bars, as well as the bottom concrete surface, were recorded using conventional strain gages, as well as using a Brillouin Optical Time Domain Analysis (BOTDA) optical fibers sensing system. Key findings of this study are summarized below:

1. The application of the hybrid FRP resulted in a much-improved flexural response of the beams as compared to the control beam both in terms of peak loads and maximum strains. A comparison of flexural responses of the strengthened beams suggested that the U-shaped pattern resulted in much higher sustained peak loads and bottom steel strains. Nonetheless, both beams were able to sustain bottom longitudinal steel strains much beyond their yield capacities.
2. Two schemes for the application of BOTDA sensing was adopted as an alternative to conventional strain gages, namely, end and spot clamping. Optical fibers mounted using only end clamps could not capture any steel strains. The ability of optical fibers to measure steel strain was greatly influenced by the presence of bottom flexural cracks. The control beam exhibited the highest number of cracks. Consequently, optical fibers failed at a very low load before the bottom steel bars yielded. The U-shaped strengthening scheme resisted the propagation of flexural cracks toward the top surface. As a result, BOTDA was able to capture steel strains beyond its yield limit.
3. Nonlinear structural analysis was performed using the computer program ATENA to replicate the experimental results. Analytical results for the control beam specimen showed a close match with corresponding experimental results mainly in terms of maximum deflection. However, the analytical peak load was slightly higher than the corresponding experimental value. Analytical responses of the strengthened beams also exhibited slightly higher peak loads than their corresponding experimental values. Unlike the control specimen, the maximum recorded deflection was much lower than their corresponding experimental values.

## Figures and Tables

**Table 1:** Test matrix and strengthening scheme.

Beam ID	Hybrid FRP Layers	Strengthening Pattern
B-CON	N/A	N/A
B-01	4	Bottom face only
B-02	4	U-shaped pattern

**Table 2:** Mechanical properties of steel reinforcement.

Bar Type	Elastic Modulus (GPa)	Yield Stress (MPa)	Yield Strain (%)	Fracture Stress (MPa)	Fracture Strain (%)
DB12	200	520	2.7	660	17.8
RB6	220	330	1.57	480	185

**Table 3:** Concrete properties.

Material	Elastic Modulus (MPa)	Tensile Strength (MPa)	Compressive Strength (MPa)
Concrete	2.75 × 10^4^	1.98	20.4

**Table 4:** Properties of composite polymers.

FRP Type	Peak Stress (MPa)	Fracture Strain (%)	Bond Strength (MPa)
Basalt	81	2.4	N/A
Jute	16.3	1.26	N/A
Epoxy	75	N/A	2.11

**Table 5:** Energy dissipation capacity.

Specimen ID	Energy Dissipation Capacity (kN-mm)	Change from B-CON (%)
B-CON	1097	0
B-01	1323	+20.5
B-02	1409	+28.4

**Table 6:** Key strain gage values for bottom longitudinal steel bars.

Scheme 1800	Yielding Strain (Microns)		Maximum Strain (Microns)		Load against Maximum Strain (kN)	
	Tension	Compression	Tension	Compression	Tension	Compression
B-CON	1800	−566	4260	−4610	50	54
B-01	2000	−574	9255	−4667	56	58
B-02	1500	−642	11,035	−1961	77	74

**Table 7:** Maximum strain from BOTDA.

Specimen ID	Maximum Strain (Microns)			Peak Load (kN)
	Bottom Steel	Top Steel	Bottom Concrete	
B-CON	1128	133	1414	40
B-02	2087	165	2900	56

**Table 8:** Comparison between experimental and analytical responses.

Specimen ID	Peak Load (kN)			Maximum Deflection (mm)		
	Experimental	Analytical	Difference (%)	Experimental	Analytical	Difference (%)
B-CON	51.4	52.8	2.73	17.40	17.71	1.72
B-01	68.7	68.3	0.58	24.90	20.62	17.27
B-02	73.1	73.7	0.82	25.05	20.35	18.76

## Data Availability

The data presented in this study are available on request from the corresponding author.
